# Changes in choroidal thickness and optic nerve head morphology after filtering surgery: nonpenetrating deep sclerectomy versus trabeculectomy

**DOI:** 10.1186/s12886-019-1031-3

**Published:** 2019-01-21

**Authors:** Aymeric Bouillot, Alexandra Pierru, Esther Blumen-Ohana, Emmanuelle Brasnu, Christophe Baudouin, Antoine Labbé

**Affiliations:** 1 Department of Ophthalmology III, Quinze-Vingts National Ophthalmology Hospital, DHU Sight Restore, 28 rue de Charenton, 75012 Paris, France; 2 Department of Ophthalmology, Ambroise Paré Hospital, AP-HP, University of Versailles Saint-Quentin-en-Yvelines, Versailles, France; 3 Department of Ophthalmology II, Quinze-Vingts National Ophthalmology Hospital, Paris, France; 4 CHNO des Quinze-Vingts, DHU Sight Restore, INSERM-DHOS CIC, 28 rue de Charenton, 75012 Paris, France; 5 INSERM, U968, F-75012 Paris, France; 6 Sorbonne Universités, UPMC Univ Paris 06, INSERM, CNRS, Institut de la Vision, 17 rue Moreau, 75012 Paris, France; 7 CNRS, UMR_7210, F-75012 Paris, France

**Keywords:** Trabeculectomy, Nonpenetrating deep sclerectomy, Choroid, Lamina cribrosa, OCT-EDI

## Abstract

**Background:**

The purpose of this study was to evaluate the changes in choroidal thickness and lamina cribrosa position after nonpenetrating deep sclerectomy (NPDS) and trabeculectomy.

**Methods:**

Twenty-three eyes with glaucoma that required filtering surgery were included (12 NDPS and 11 trabeculectomies) in this prospective observational study. OCT-enhanced depth imaging (OCT-EDI) was used to measure choroidal thickness, prelaminar tissue thickness and lamina cribrosa position before and 7 days and 1 month after surgery. All results are shown as median (interquartile range values).

**Results:**

Intraocular pressure (IOP) was significantly lower 1 week after surgery than at baseline (7 (6/10) mmHg vs. 21 (18/26) mmHg; *p* < 0.001) with a mean 64% decrease. IOP remained significantly lower at 1 month with a 55% mean decrease as compared to baseline (10 (8/12) mmHg; *p* < 0.001). One week after surgery, the subfoveolar choroidal thickness (SFCT) significantly increased (372 (306/523) μm vs. 317 (227/413) μm; *p* = 0.04) and the prelaminar tissue (PLT) was significantly thicker (269 (162/360) μm vs. 138 (87/268) μm; *p* = 0.02) as compared to preoperative measurements. These changes were not statistically significant at one month. There were no differences concerning these parameters between the NPDS and trabeculectomy groups. During the first week, the SFCT increase was correlated with IOP reduction (r = − 0.41; *p* = 0.04).

**Conclusions:**

OCT-EDI allowed the visualization of structural changes at the level of the optic nerve and choroidal vascularization during acute IOP changes. No difference was observed between NPDS and trabeculectomy concerning these structural modifications.

## Background

The lamina cribrosa seems to play a major role in glaucoma pathophysiology. This structure is composed of piles of connective tissue layers that are fenestrated by pores in which bundles of retinal ganglion cell (RGC) axons pass [1]. The lamina cribrosa is a dynamic structure that is modified by chronic intraocular pressure (IOP) elevation [2]. IOP rise may compress the collagen plates and affect the laminar pore area [3], with mechanical stress to RGC axons and consequently RGC apoptosis and death [4]. Although a transient IOP elevation does not seem to change lamina cribrosa position [5], this structure is thinner and more posterior compared to the Bruch membrane opening (BMO) in glaucoma patients [6, 7]. Therefore, the impact of high IOP on lamina cribrosa seems to be a gradual process.

Vascular impairment in the optic nerve head also seems to be a relevant component in the pathogenesis of glaucomatous optic neuropathy [8]. Local vasospasm, blood hypertension and nocturnal blood hypotension are associated with glaucomatous neuropathy progression [9]. Using laser Doppler flowmetry, several studies reported optic nerve head (ONH) blood flow reduction in glaucomatous eyes [10]. Hypoperfusion may induce RGC axon ischemia and oxidative stress [11]. Most recently, using optical coherence tomography angiography, some authors, including our group, detected reduced ONH blood vessel density in glaucoma patients [12, 13]. Similarly, a decrease in choroidal blood flow has been described in glaucoma patients [14], but choroidal thickness has been reported not to be affected in glaucoma [15–17].

Spaide et al. first described enhanced depth imaging optical coherence imaging (OCT-EDI) using the inverted image obtained when the device is closer to the eye [18]. This method provides higher image resolution on layers located under the retinal pigment epithelium. It is now possible to visualize the lamina cribrosa anterior surface and the scleral inner surface and then to assess choroidal thickness and lamina cribrosa position [19, 20].

IOP remains the main target of glaucoma treatment and, in case of glaucomatous neuropathy progression despite maximally tolerated medications, filtering surgery can be indicated. Although trabeculectomy is considered as the standard surgical procedure to reduce IOP in glaucoma patients, nonpenetrating deep sclerectomy (NPDS) may provide comparable IOP reduction with the advantage of a lower rate of postoperative complications, particularly those related to immediate postoperative hypotony [21, 22]. Previous studies suggested that the IOP decrease following trabeculectomy causes choroidal thickening [23–27] and lamina cribrosa anterior displacement [28, 29]. To our knowledge, modifications of choroidal thickness and optic nerve head induced by NPDS have not been assessed yet. Therefore, the aim of the present study was to evaluate the changes in choroidal thickness and lamina cribrosa position after NPDS and trabeculectomy.

## Methods

This was a prospective observational study of consecutive patients undergoing glaucoma filtering surgery at Quinze-Vingts National Ophthalmology Hospital, Paris, France, between March 2014 and August 2015. All participants involved were informed of the purpose of this study and written consent was obtained. This study has been approved by the Ethics Committee of the French Society of Ophthalmology (IRB 00008855 Société Française d’Ophtalmologie IRB#1). Filtering surgery was provided to patients with uncontrolled IOP after maximum glaucoma medications and functional progression confirmed by visual field examination. According to European Glaucoma Society guidelines [30], we performed NPDS for primary open-angle glaucoma (POAG) patients and trabeculectomy for primary angle-closure glaucoma (PACG). Glaucoma was diagnosed on the basis of clinical examination, retinal nerve fiber layer analyses using Spectral Domain Optical Coherence Tomography (SD-OCT) and visual field examination. The exclusion criteria were age under 18, history of intraocular inflammation or any retinal abnormalities, history of nonglaucomatous optic neuropathy, optic nerve drusen, history of previous intraocular surgery (except cataract or refractive surgery) or laser therapy.

All patients underwent filtering surgery (trabeculectomy or NPDS according to the individual physician’s judgment and experience) with mitomycin-C (MMC) application followed in the postoperative period by a treatment with tobramycin/dexamethasone and unpreserved indomethacin eyedrops for at least 2 months after surgery. All glaucoma medications were stopped after surgery.

At baseline, all patients underwent ophthalmological examinations including best corrected visual acuity (BCVA), slit-lamp biomicroscopy, gonioscopy, applanation tonometry and dilated funduscopy. They also underwent a central corneal thickness (CCT) measurement with ultrasound pachymetry (pachμmeter, Haag-Streit, Bern, Switzerland), an axial length measurement with partial optical coherence interferometry (IOLMaster 500; Carl Zeiss Meditec, La Jolla, CA, USA) and a visual field examination (SITA standard 24–2, Humphrey Field Analyzer – HFA II; Carl Zeiss Meditec). Systolic blood pressure (SBP) and diastolic blood pressure (DBP) were also measured preoperatively on the upper arm by an automated device. Mean blood pressure (mBP) was calculated as the DBP plus one-third the difference between SBP and DBP. The ocular perfusion pressure (OPP) was calculated as 2/3rd of mBP minus IOP [31].

Optic nerve head and macular imaging were performed using Spectralis® SD-OCT (Heidelberg Engineering GmbH, Heidelberg, Germany) at baseline, 1 week and 1 month after surgery. The image registration feature of the Heidelberg Spectralis® device ensured that the same area was scanned each time. OCT images were taken by the same experienced operator, with enhanced depth imaging (EDI), allowing better visualization of the anterior laminar surface, the choroid and especially the choroid–sclera interface. All measurements were taken within a limited time (8:00 am to 11:00 am) to reduce the influence of diurnal variation of choroidal thickness [32]. Four B-scans were made up during each eye examination: [1] a vertical scan closest to the optic nerve head center, which did not include central artery or vein of the retina; [2] a 360-degree 3.4-mm-diameter peripapillary circle scan centered on the optic disc; [3] a vertical macular scan centered on the fovea and [4] a horizontal macular scan centered on the fovea. The resulting images were viewed and analyzed with the Eye Explorer software (v 1.7.1.0; Spectralis®, Heidelberg Engineering GmbH, Germany). Each measurement was taken by the same operator that was masked for patient status, using the software’s caliper tool.

**Fig. 1:**
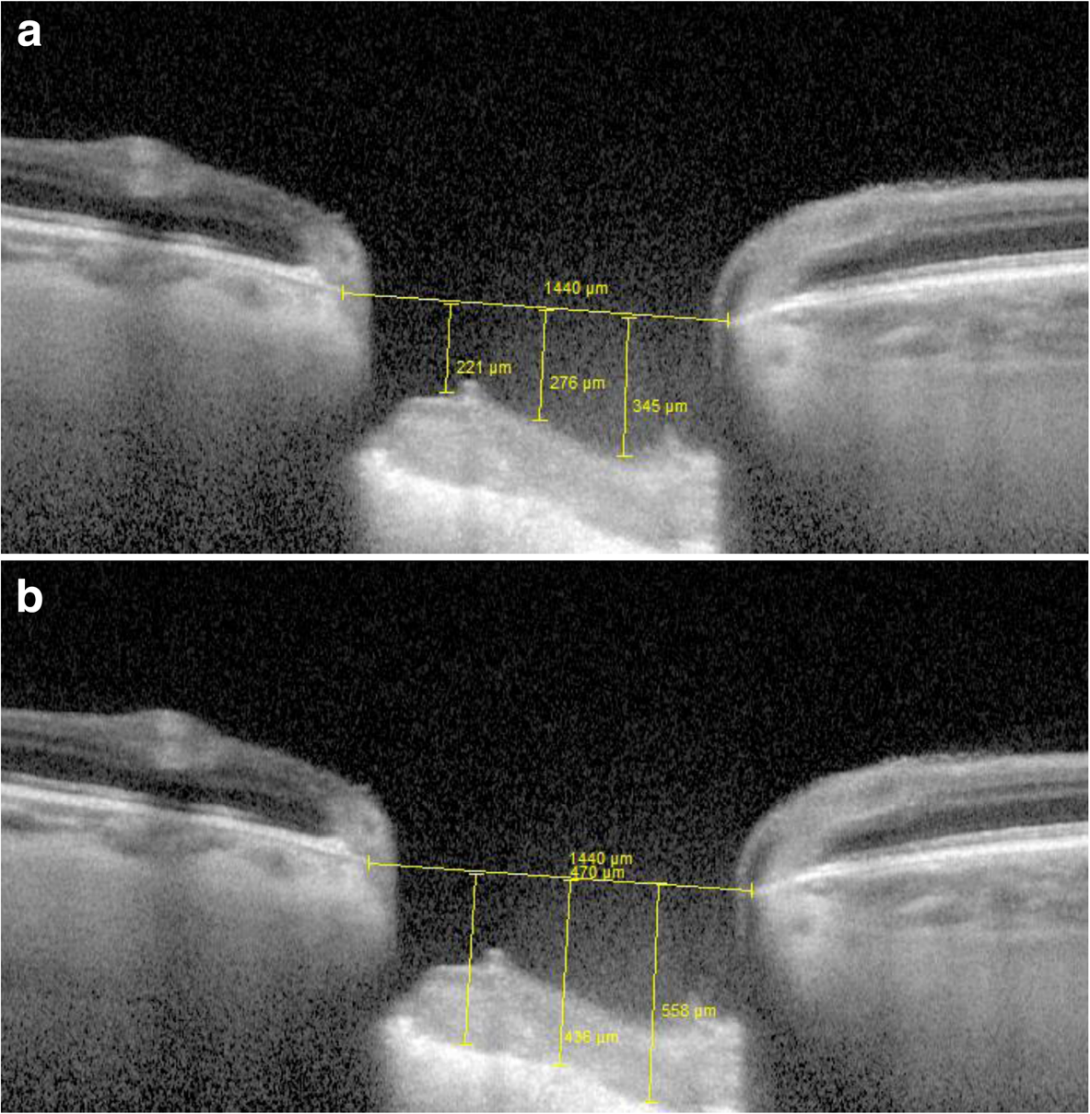
OCT-EDI vertical scan through optic nerve head. Example of cup (**a**) and lamina cribrosa depth (**b**) measurements

On the vertical optic nerve head scans, a reference line was drawn connecting the two terminations of the Bruch membrane. Three equidistant points were placed on this line. The distances from the reference line to the prelaminar tissue anterior surface were measured at these three points. The mean of the three measurements was defined as the cup (Fig. 1a). The distances from the reference line to the lamina cribrosa anterior surface were also measured. The mean of these measurements was defined as the lamina cribrosa depth (LCD) (Fig. 1b). Prelaminar tissue thickness (PLT) was considered as the difference between cup and LCD.

**Fig. 2:**
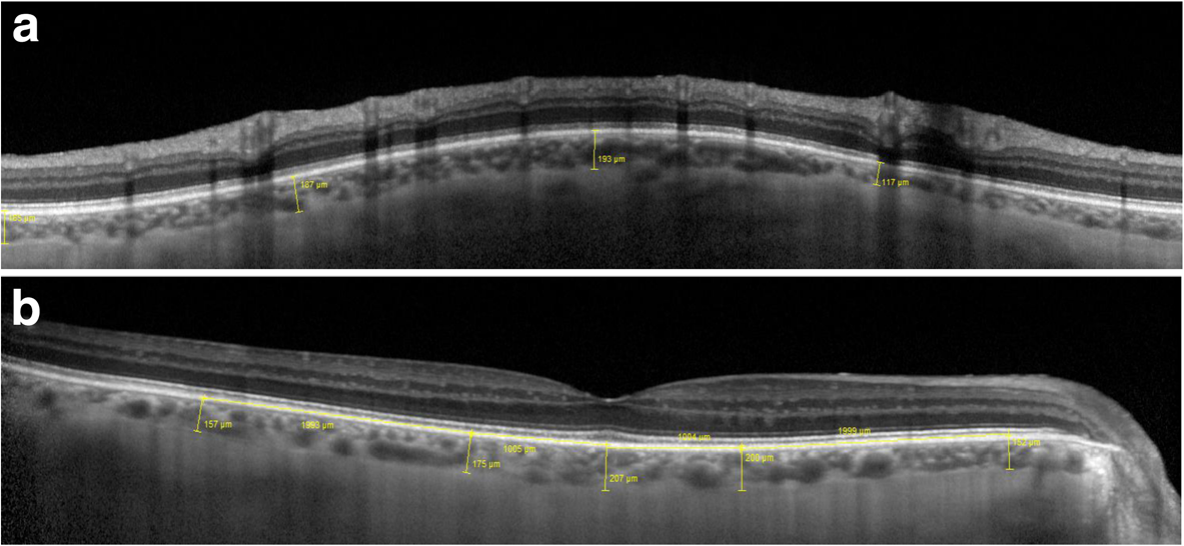
**a** OCT-EDI peripapillary circle scan. Example of peripapillary choroidal thickness measurement. **b** OCT-EDI horizontal scan centered on the fovea. Examples of subfoveolar choroidal thickness, measured at 1000 μm and 3000 μm in nasal and temporal areas

On peripapillary scans, the choroid was manually segmented and measured between the Bruch membrane hyperreflective line and the hyperreflective line of the inner surface of the sclera (Fig. 2a). The choroidal thickness was assessed in the temporal, nasal, superior and inferior sectors. The mean of these measurements was defined as mean peripapillary choroidal thickness (PCT). On vertical and horizontal macular scans, the choroidal thickness was measured using the same anatomic boundaries. Subfoveolar choroidal thickness (SFCT) was measured. We also measured choroidal thickness at 1000 μm and 3000 μm from the fovea in the nasal, temporal, superior and inferior sectors (Fig. 2b). The mean macular choroidal thickness (MCT) was defined as the mean of these nine measurements (the subfoveolar thickness plus two measurements in each quadrant). All measurements were taken at baseline, 1 week and 1 month after surgery.

Statistical analyses were performed using the SPSS software package version 20.0 (SPSS Inc., Chicago, IL, USA). Changes in IOP, SFCT, PCT and MCT were defined as baseline values minus first week or one month values. The nonparametric Mann-Whitney test was used to compare the continuous data and the χ [2] test was used to compare categorical data. As the data distribution was not normal, Spearman’s correlation was used to correlate any quantitative parameters. Statistical significance was *p* ≤ 0.05. All results are shown as median (interquartile range values).

## Results

Twenty-three eyes of 23 glaucomatous patients requiring filtering surgery were included in this study. There were 16 men (69.5%) and seven (30.5%) women. The patients’ median age was 60 (52/74) years. At baseline, demographic and clinical characteristics were comparable between the NPDS and trabeculectomy groups (Table 1).

**Table 1:** Demographic and clinical characteristics of patients at baseline and after surgery, comparison between NPDS and trabeculectomy groups

Variables	Overall study population	NPDS group	Trabeculectomy group	*p*
Number of patients	23	12	11	–
Age (years)	60 (52/74)	58 (52/73.25)	63 (53/76)	0.64
Gender: M / F	16/7	8/4	8/3	0.75
Axial length (mm)	23.63 (23.26/23.9)	23.61 (23.22/24.33)	23.64 (23.26/23.8)	0.93
Spherical equivalent (D)	0.00 (−1,75/1)	0.00 (−1.75/1.19)	−1 (− 1.5/1)	1
Central corneal thickness (μm)	547 (514/565)	548.5 (506/563.75)	535 (514/566)	0.9
Visual field MD (dB)	−13.72 (−19.32/−5.01)	−11.06 (− 17.54/−3.96)	− 14.9 (−21.84/− 5.01)	0.45
Number of topical medications	3 (3/3)	3 (3/3)	3 (3/4)	0.49
SBP (mmHg)	130 (120/135)	130 (120/134.5)	130 (123/140)	0.45
DBP (mmHg)	80 (75/80)	78.5 (72/80)	80 (75/80)	0.73
mBP (mmHg)	95 (93.3/98)	95.35 (90.82/96.92)	95 (93.3/100)	0.62
OPP (mmHg)	52.9 (43.3/74)	64.15 (45.97/74.75)	47 (36.5/64)	0.14
IOP, preoperative (mmHg)	21 (18/26)	21.5 (17.25/24)	20 (18/36)	0.44
IOP, 1 week (mmHg)	7 (6/10)	8.5 (7/13)	6 (4/10)	**0.04**
IOP, 1 month (mmHg)	10 (8/12)	10.5 (8/12)	10 (8/12)	0.66

*MD* mean deviation, *SBP* Systolic blood pressure, *DBP* Diastolic blood pressure, *mBP* Mean blood pressure, *OPP* Ocular perfusion pressure, *IOP* Intraocular pressure, P: comparison between NPDS and trabeculectomy groups

The mean IOP was significantly lower 1 week after surgery than at baseline (7 (6/10) mmHg vs. 21 (18/26) mmHg; *p* < 0.001) with a mean 64% decrease. IOP remained significantly lower at 1 month with a 55% mean decrease as compared to baseline (10 (8/12) mmHg; *p* < 0.001) (Table 1). IOP was significantly lower in the trabeculectomy group at 1 week (6 (4/10) mmHg vs. 8.5 (7/13) mmHg; *p* = 0.04), but the difference was no longer statistically significant at 1 month (*p* = 0.66) (Table 1).

Table 2 summarizes the main parameters assessed after surgery. The prelaminar tissue (PLT), subfoveolar choroidal thickness (SFCT) and mean macular choroidal thickness (MCT) increased significantly 1 week after surgery (*p* = 0.02, *p* = 0.04 and *p* = 0.04, respectively). However, this difference was not significant at 1 month (Table 2). There was no significant difference for the lamina cribrosa depth (LCD) and the mean peripapillary choroidal thickness (PCT) after surgery. PLT was thicker in the trabeculectomy group at baseline (234 (138/279) μm vs. 90.5 (58.5/201.25) μm; *p* = 0.01), 1 week (360 (276/379) μm vs. 163 (104.75/247.25) μm; *p* = 0.002) and 1 month (272 (191/347) μm vs. 152.5 (94.25/227) μm). Nevertheless, there was no difference between the two groups concerning PLT change after 1 week (*p* = 0.19) or 1 month (*p* = 0.66). Similarly, there was no difference between NPDS and trabeculectomy concerning LCD, PCT, SFCT and MCT at any time of the study*.*

**Table 2:** Clinical measurements at baseline and after surgery

	Preoperative	1 Week	1 Month	*p* ^*1*^	*p* ^*2*^	*p* ^*3*^
LCD (μm)	544 (438/730)	516 (362/683)	571 (358/698)	0.43	0.64	0.77
PLT (μm)	138 (87/268)	269 (162/360)	206 (135/283)	**0.02**	0.47	0.16
PCT (μm)	183 (108.75/238.25)	217.75 (171.75/294)	179.75 (161.25/251.75)	0.1	0.31	0.42
SFCT (μm)	317 (227/413)	372 (306/523)	314 (267/578)	**0.04**	0.38	0.16
MCT (μm)	265.33 (203.11/406.22)	280.8 (253.1/477.4)	232.64 (207.09/452.91)	**0.04**	0.09	0.96

*LCD* Lamina cribrosa depth, *PLT* Prelaminar tissue thickness, *PCT* Mean peripapillary choroidal thickness, *SFCT* Subfoveolar choroidal thickness, *MCT* Mean macular choroidal thickness, p^1^: comparison between baseline and week 1; p^2^: comparison between week 1 and 1 month; p^3^: comparison between baseline and 1 month

At baseline, LCD was correlated with age (Spearman r = − 0.52; *p* = 0.01) and systolic blood pressure (Spearman r = − 0.47; *p* = 0.02) (Table 3). PCT was correlated with age (Spearman r = − 0.60; *p* = 0.002). SFCT was correlated with age (Spearman r = − 0.49; *p* = 0.018) and axial length (Spearman r = − 0.50; *p* = 0.016). MCT was correlated with age (Spearman r = − 0.56; *p* = 0.005) and axial length (Spearman r = − 0.53; *p* = 0.009) (Table 4). Tables 3 and 4 show the relationships between LCD, PLT, PCT, SFCT, MCT and clinical characteristics at baseline.

**Table 3:** Relationships between lamina cribrosa depth (LCD), prelaminar tissue thickness (PLT) and clinical characteristics at baseline

Parameters		LCD (μm)		PLT (μm)
	Spearman r	*p*	Spearman r	*p*
Age (years)	−0.52	**0.01**	0.08	0.71
Axial length (mm)	−0.04	0.85	0.08	0.71
Spherical equivalent (mm)	−0.06	0.79	− 0.02	0.92
Central corneal thickness (μm)	−0.09	0.68	0.23	0.28
Visual field MD (dB)	−0.18	0.40	0.22	0.30
Number of topical medications	−0.24	0.26	0.26	0.24
SBP (mmHg)	−0.47	**0.02**	0.30	0.16
DBP (mmHg)	0.28	0.19	0.13	0.56
mBP (mmHg)	0	0.99	0.17	0.44
OPP (mmHg)	−0.02	0.93	−0.34	0.12
IOP (mmHg)	0.27	0.22	0.18	0.41

*MD* mean deviation, *SBP* Systolic blood pressure, *DBP* Diastolic blood pressure, *mBP* Mean blood pressure, *OPP* Ocular perfusion pressure

**Table 4:** Relationships between mean peripapillary choroidal thickness (PCT), subfoveolar choroidal thickness (SFCT), mean macular choroidal thickness (MCT) and clinical characteristics at baseline

Parameters		PCT (μm)		SFCT (μm)		MCT (μm)
	Spearman r	*p*	Spearman r	*p*	Spearman r	*p*
Age (years)	−0.60	**0.002**	−0.49	**0.018**	−0.56	**0.005**
Axial length (mm)	−0.37	0.08	−0.50	**0.016**	−0.53	**0.009**
Spherical equivalent (mm)	0.12	0.60	0.19	0.39	0.16	0.47
Central corneal thickness (μm)	0.26	0.23	0.29	0.18	0.25	0.25
Visual field MD (dB)	0.06	0.77	−0.02	0.94	- 0.02	0.92
Number of topical medications	−0.17	0.44	−0.03	0.91	−0.07	0. 74
SBP (mmHg)	−0.26	0.23	- 0.02	0.94	−0.07	0.75
DBP (mmHg)	0.38	0.07	0.32	0.14	0.32	0.14
mBP (mmHg)	0.21	0.35	0.26	0.22	0.26	0.22
OPP (mmHg)	−0.04	0.86	- 0.09	0.69	- 0.07	0.75
IOP (mmHg)	−0.07	0.77	−0.13	0.57	−0.15	0.48

*MD* mean deviation, *SBP* Systolic blood pressure, *DBP* Diastolic blood pressure, *mBP* Mean blood pressure, *OPP* Ocular perfusion pressure

**Fig. 3:**
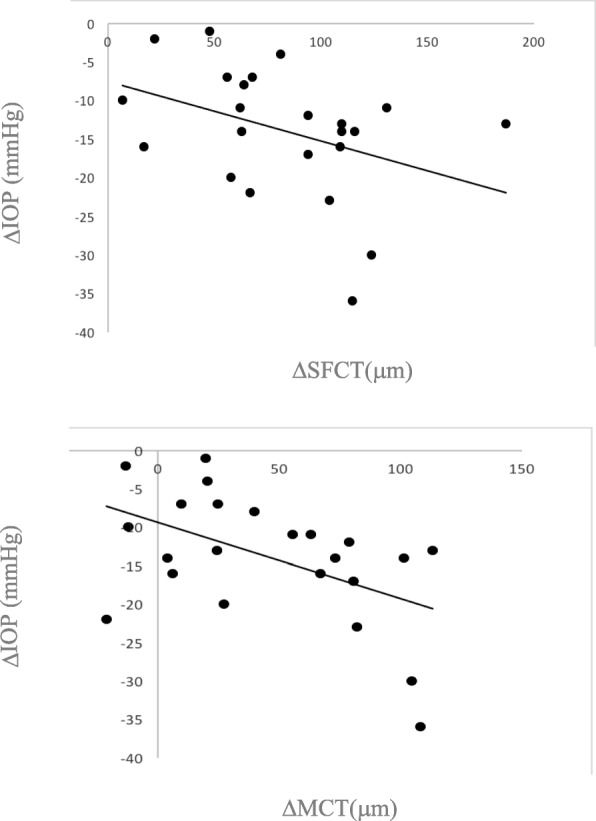
Relationships between IOP decrease and changes in subfoveolar choroidal thickness (SFCT) and mean macular choroidal thickness (MCT) 1 week after surgery. a: ΔIOP was correlated with ΔSFCT, Spearman r = − 0.41; *p* = 0.04. b: ΔIOP was correlated with ΔMCT, Spearman r = − 0.46; *p* = 0.02

One week after surgery, the changes in SFCT and MCT were negatively correlated with the change in IOP (Spearman r = − 0.41; *p* = 0.04 and Spearman r = − 0.46; *p* = 0.02), (Fig. 3).

**Fig. 4:**
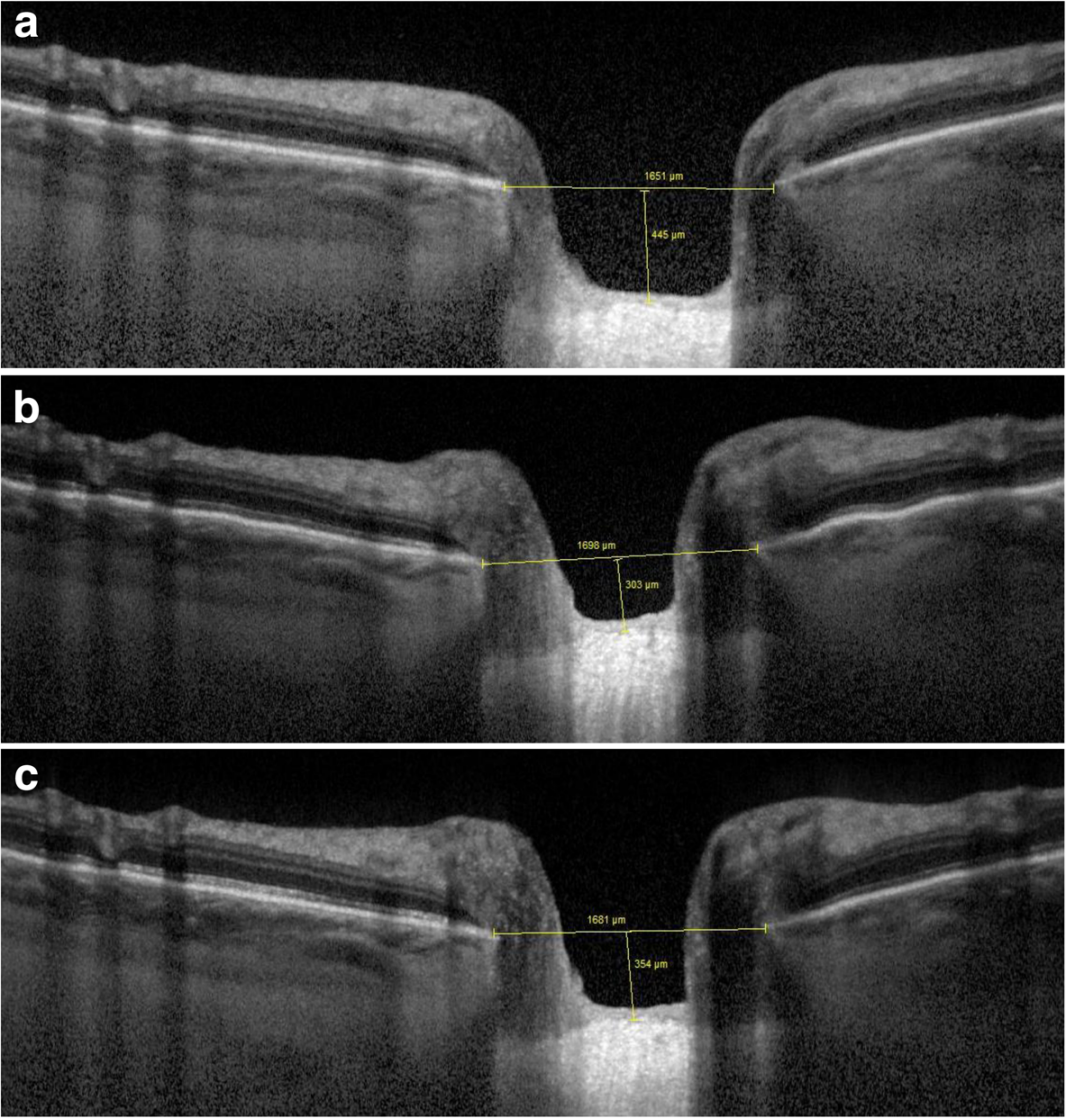
Example of morphological changes in optic nerve head after NPDS. Preoperatively, (**a**) lamina cribrosa depth was 445 μm and prelaminar tissue thickness was 42 μm. At 1 week (**b**), lamina cribrosa depth was 303 μm and prelaminar tissue thickness was 53 μm. At 1 month (**c**), lamina cribrosa depth was 354 μm and prelaminar tissue thickness was 53 μm

**Fig. 5:**
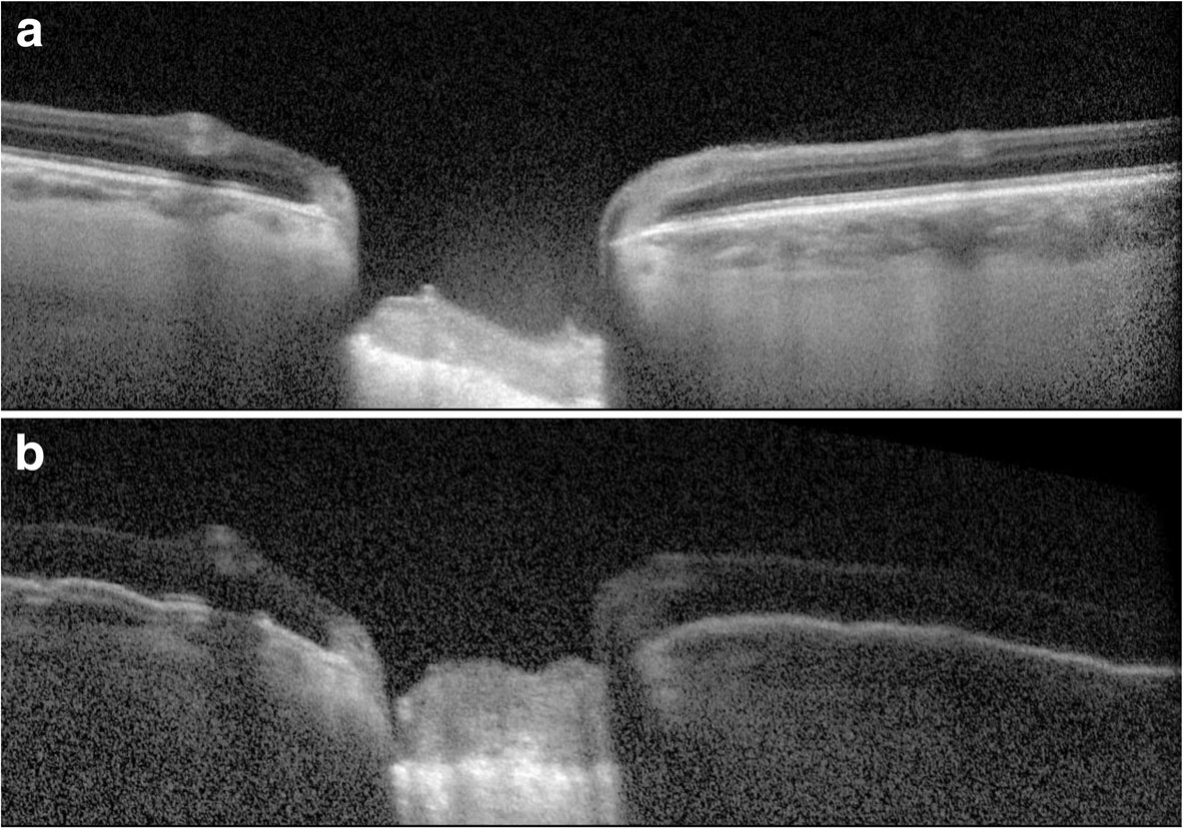
Example of morphological changes in optic nerve head after trabeculectomy. Preoperatively, (**a**) lamina cribrosa depth was 483 μm and prelaminar tissue thickness was 194 μm. At 1 week, (**b**) lamina cribrosa depth was 360 μm and prelaminar tissue thickness was 360 μm

**Fig. 6:**
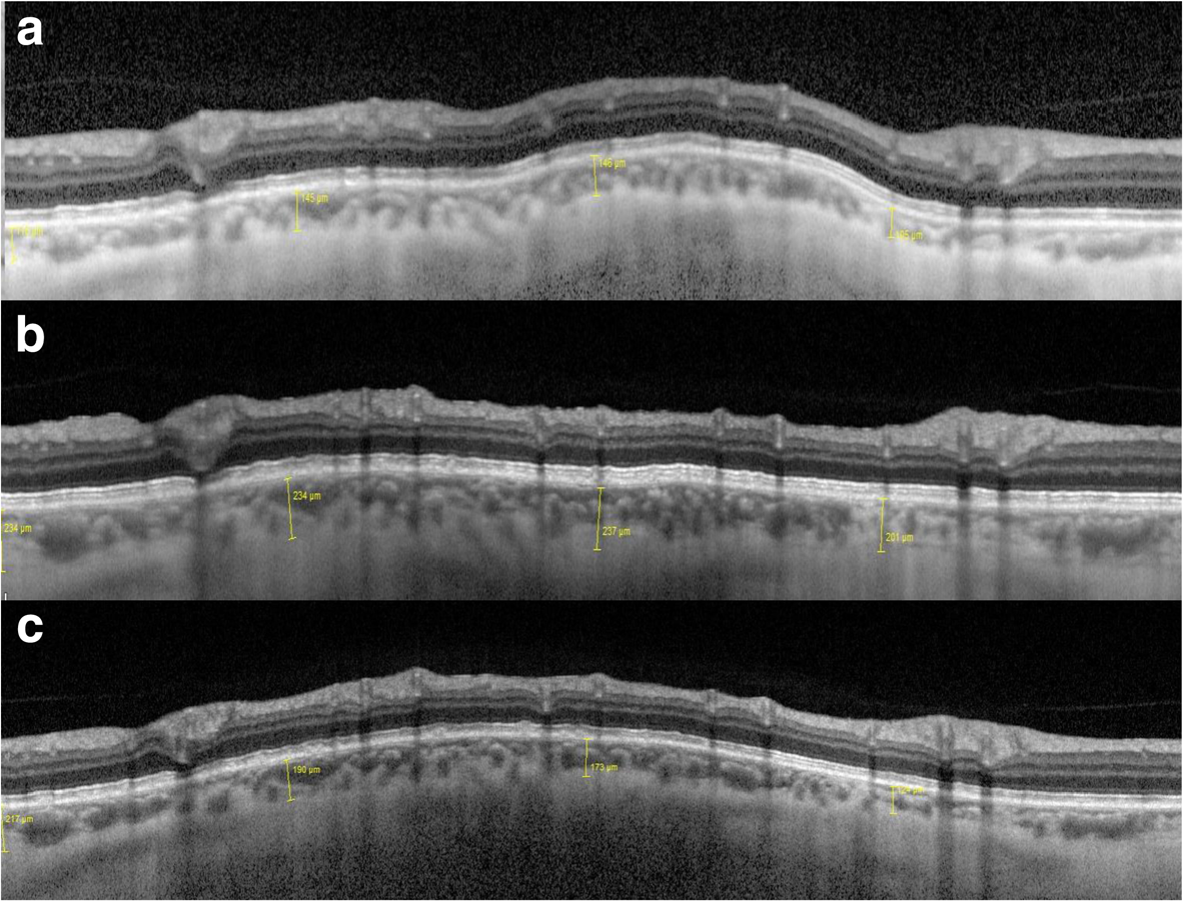
Example of morphological changes in peripapillary choroidal thickness after NPDS. Preoperatively, (**a**) PCT was 127.75 μm. At 1 week, (**b**) PCT was 226.5 μm. At 1 month, (**c**) PCT was 176 μm

**Fig. 7:**
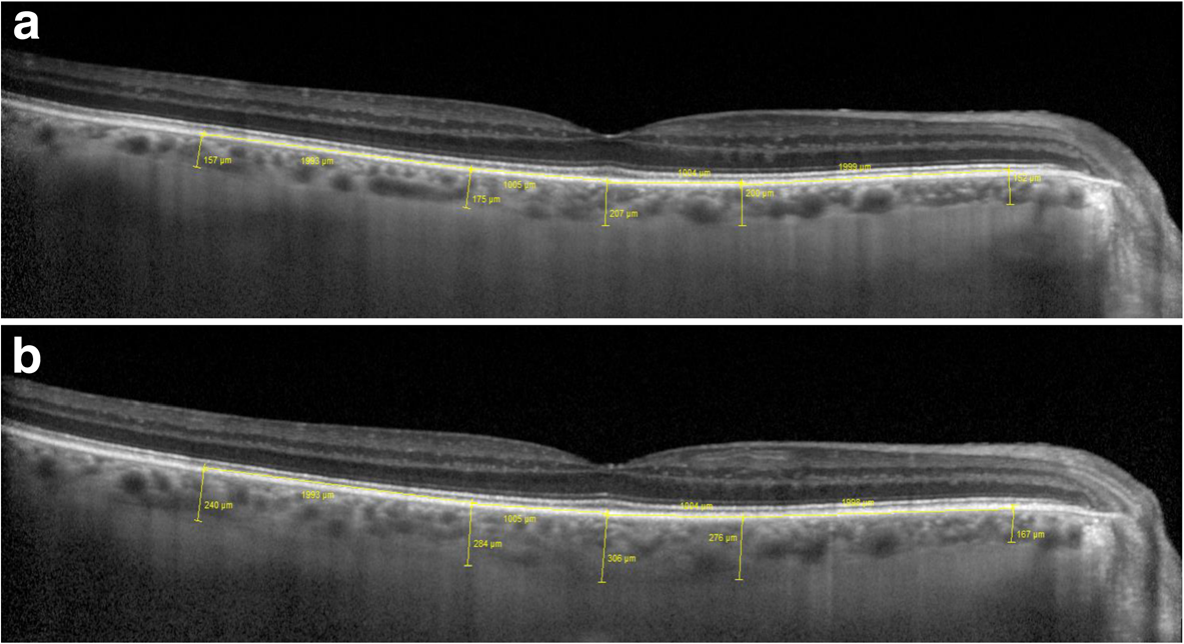
Example of morphological changes in macular choroidal thickness after NPDS. Preoperatively, (**a**) SFCT was 172 μm, MCT was 183.55 μm. At 1 week, (**b**) SFCT was 306 μm, MCT was 267.9 μm

Figures 4, 5, 6 and 7 show examples of lamina cribrosa shape and choroidal changes after filtering surgery (Figs. 4, 5, 6 and 7).

## Discussion

Excepting angle anatomy, the two groups were comparable at baseline. This was consistent with Beijing eye study results, angle opening degree seems not the affect choroidal thickness [33]. This also corroborates with Lee et al. findings, angle anatomy seems not to affect lamina cribrosa position [34]. Then, we consider that post operative differences between the two groups are mainly due to IOP reduction difference during the first week.

Consistent with previous studies analyzing similar parameters after trabeculectomy [23, 26, 35], we found overall choroidal thickening 1 week after surgery. This finding was not significant at 1 month and may correspond to an early stage of hypotony maculopathy during the first postoperative days. Since we did not find any difference concerning choroidal thickness between the two groups, we hypothesize that early hypotony maculopathy risk is not lower after NPDS as compared to that of trabeculectomy. Choroidal thickening is related to IOP reduction, but its exact pathophysiology remains uncertain. According to Saeedi et al., choroidal thickness increases as the force of IOP on the choroid decreases [25] and as the axial length decreases due to scleral relaxation [36]. According to Kara et al., choroidal thickening is related to an OPP increase induced by IOP reduction [24]. This corresponds to the retrobulbar blood flow rising observed after trabeculectomy [37]. Nickla and Wallman presumed that the choroidal thickness increase stemmed from two mechanisms [38]: first the synthesis of osmotically active proteoglycans, which help pull water into the choroid; second, to the increase in the size or number of the choriocapillaris vessels. Recently, Zhang et al. showed that the choroidal thicknening observed when decreasing IOP occurred both in the large choroidal vessels and the interstitium of the choroid [39]. This corroborates with the osmotic mechanism hypothesis. Mermoud et al. assessed the efficacy and postoperative complications of NPDS. They observed a lower rate of choroidal detachement after NPDS than after trabeculectomy (5% vs. 20%; *p* = 0.05) [40]. This complication, which usually occurs during the first weeks after filtering surgery seems to be correlated with lower IOP and may impair visual function [41, 42]. Given that choroidal thickening is directly related to IOP changes, the absence of very low IOP in the trabeculectomy group in our study might explain the absence of a difference between the two groups.

One week after surgery, we also found a significant PLT increase. This result can be compared to that reported by Lee et al., who observed significant PLT thickening at postoperative month 6 (95.77 ± 40.97 μm at baseline vs. 101.71 ± 42.06 μm postoperatively; *p* = 0.048) [29]. However, in the present study, this change was not correlated with IOP reduction and there were no significant differences between NPDS and trabeculectomy groups. Explanation for this thickening remains uncertain. Previous studies hypothesized that the vessel size could increase due to a rise in ocular blood flow [37]. Reis et al. also suggested that this modification is caused by size changes in RGC axons and astrocytes after IOP reduction [28]. Our results corroborate the findings of Agoumi et al., who observed that a quick IOP rise induced prelaminar tissue compression [5].

We did not find any significant difference concerning lamina cribrosa position before and after filtering surgery. Moreover, there was no difference between NPDS and trabeculectomy groups for this parameter. In a study evaluating changes in lamina cribrosa position after trabeculectomy in glaucoma patients, Reis et al. reported anterior lamina cribrosa displacement. However, their results were only significant at 6 months after surgery [28]. Since the lamina cribrosa is mainly composed of collagen [43, 44], it can be hypothesized that it is less compliant to quick IOP reduction, as observed in the early postoperative period, than the prelaminar tissue, which is composed of vascular and nervous elements [45]. This may explain the lack of laminar displacement in our study conducted during the 1st month after surgery. However, we cannot exclude that laminar displacement may occur later after surgery. Furthermore, OCT-EDI allows the analysis of the anterior surface of the lamina cribrosa, but visualization of the posterior surface of the lamina cribrosa remains difficult with this technique. Consequently, an overall displacement of the lamina cribrosa or a thickening of this structure could not be differentiated. The impact of an anterior lamina cribrosa displacement on glaucomatous neuropathy remains unclear. The axoplasmic transport obstruction previously described could be related to the signifiant pressure gradient on RGC axons as they pass through the lamina cribrosa. A chronic IOP elevation may raise this pressure gradient, impacting RGC axons and causing disease progression [46]. Anterior laminar displacement induced by filtering surgery may correspond to a reversal of the mechanical stress on RGC axons. The prelaminar tissue thickening might correspond to an increase in blood flow and a shift in axoplasmic fluid induced by this decompression.

The possibility of a loss of central vision, or “wipe-out syndrome,” has been described after trabeculectomy in patients with advanced glaucomatous optic neuropathy. In the studies that evaluated the rate of severe loss of central vision after trabeculectomy, the prevalence of this complication has been reported in 0–7.7% of these patients [41, 42, 47], and it might be related to profound hypotony during the first postoperative days [42]. According to Law et al., patients with high preoperative IOP and a sudden substantial IOP reduction are most likely to undergo this complication [41]. To our knowledge, this sudden loss of central vision has not been described after NPDS [48]. The reversal of the pressure gradient on RGC axons might be involved in wipe-out syndrome pathophysiology with the sudden axon decompression being noxious and inducing cell death. This physiopathological mechanism seems to be similar to the one described as free radical-mediated reperfusion injury. Reversal of ischemia provides acute free radical release with a chain reaction and structural changes in endothelial cells [49]. Vessel permeability rises and leads to intersitial edema, which can aggravate ischemia by a compressive mechanism. In the present study, there was no difference in prelaminar tissue thickening between the trabeculectomy and the NPDS groups. This may suggest that decompression risk is not lower after NPDS despite a lower IOP reduction. In the present study, or serous choroidal detachment during the follow up; consequently, the lack of a difference between the two groups concerning prelaminar tissue may be related to the absence of severe hypotony.

This study has some limitations including the absence of regression analysis and the small number of patients analyzed. Although, the number of patients in the trabeculectomy group was smaller than previous studies, including NPDS population it is larger than three of the four studies analyzing these parameters after trabeculectomy [23, 25, 35]. Moreover, the results after NPDS are coherent and correlated with a lower IOP reduction during the 1st postoperative week. Nevertheless, some differences between NPDS and trabeculectomy groups might remain undetected because of a lack of power due to the small number of eyes included in each group. Secondly, we measured the changes in choroid and optic nerve head that occurred during the 1st month after surgery. Even if it would have been interesting to analyze the changes after a longer period following surgery, previous similar studies found no difference between 1 month and later results (3–6 months) [25, 28, 29]. Thirdly, each measurement was taken manually using the Eye Explorer software. This technique has shown good interobserver repeatability [18, 19] and to our knowledge, there is currently no automated software to measure these parameters automatically. Finally, as these structural changes might also be related to vascular changes, it would be interesting in future studies to assess vessel density modifications using OCT-angiography.

To our knowledge, this study is the first to evaluate choroidal thickness, prelaminar tissue thickness and lamina cribrosa position changes after NPDS, and to compare the results to the effects of trabeculectomy. OCT-EDI allowed us to visualize the choroidal thickness increase and prelaminar tissue thickening during acute IOP changes following filtering surgery. These changes were greater during the 1st week after surgery and correlated with IOP reduction. No difference was observed between NPDS and trabeculectomy concerning these structural modifications. Such evaluations using a modern OCT technology adapted to deep tissues and structures may help better understand certain severe complications of filtering surgery such as the wipe-out syndrome and hypotony maculopathy.

## Abbreviations

BCVA: Best Corrected Visual Acuity
BMO: Bruch Membrane Opening
CCT: Central Corneal Thickness
DBP: Diastolic Blood Pressure
EDI: Enhanced Depth Imaging
IOP: Intraocular Pressure
LCD: Lamina Cribrosa Depth
mBP: Mean Blood Pressure
MCT: Mean Macular Choroidal Thickness
MMC: Mitomycin-C
NPDS: Nonpenetrating Deep Sclerectomy
OCT-EDI: Enhanced Depth Imaging Optical Coherence Imaging
ONH: Optic Nerve Head
OPP: Ocular Perfusion Pressure
PACG: Primary Angle-closure Glaucoma
PCT: mean Peripapillary Choroidal Thickness
PLT: Prelaminar Tissue Thickness
POAG: Primary Open-angle Glaucoma
RGC: Retinal Ganglion Cell
SBP: Systolic Blood Pressure
SD-OCT: Spectral Domain Optical Coherence Tomography
SFCT: Subfoveolar Choroidal Thickness
